# The Male Sex Pheromone of the Butterfly *Bicyclus anynana*: Towards an Evolutionary Analysis

**DOI:** 10.1371/journal.pone.0002751

**Published:** 2008-07-23

**Authors:** Caroline M. Nieberding, Helene de Vos, Maria V. Schneider, Jean-Marc Lassance, Natalia Estramil, Jimmy Andersson, Joakim Bång, Erik Hedenström, Christer Löfstedt, Paul M. Brakefield

**Affiliations:** 1 Evolutionary Biology Group, Institute of Biology, Leiden University, Leiden, the Netherlands; 2 Zoogeographical Research Unit, Institute of Botany, Liège University, Tilman, Belgium; 3 Chemical Ecology and Ecotoxicology, Department of Ecology, Lund University, Lund, Sweden; 4 Chemistry Laboratory, Department of Natural Sciences, Mid Sweden University, Sundsvall, Sweden; University of Pretoria, South Africa

## Abstract

**Background:**

Female sex pheromones attracting mating partners over long distances are a major determinant of reproductive isolation and speciation in Lepidoptera. Males can also produce sex pheromones but their study, particularly in butterflies, has received little attention. A detailed comparison of sex pheromones in male butterflies with those of female moths would reveal patterns of conservation versus novelty in the associated behaviours, biosynthetic pathways, compounds, scent-releasing structures and receiving systems. Here we assess whether the African butterfly *Bicyclus anynana*, for which genetic, genomic, phylogenetic, ecological and ethological tools are available, represents a relevant model to contribute to such comparative studies.

**Methodology/Principal Findings:**

Using a multidisciplinary approach, we determined the chemical composition of the male sex pheromone (MSP) in the African butterfly *B. anynana*, and demonstrated its behavioural activity. First, we identified three compounds forming the presumptive MSP, namely (*Z*)-9-tetradecenol (Z9-14:OH), hexadecanal (16:Ald ) and 6,10,14-trimethylpentadecan-2-ol (6,10,14-trime-15-2-ol), and produced by the male secondary sexual structures, the androconia. Second, we described the male courtship sequence and found that males with artificially reduced amounts of MSP have a reduced mating success in semi-field conditions. Finally, we could restore the mating success of these males by perfuming them with the synthetic MSP.

**Conclusions/Significance:**

This study provides one of the first integrative analyses of a MSP in butterflies. The toolkit it has developed will enable the investigation of the type of information about male quality that is conveyed by the MSP in intraspecific communication. Interestingly, the chemical structure of *B. anynana* MSP is similar to some sex pheromones of female moths making a direct comparison of pheromone biosynthesis between male butterflies and female moths relevant to future research. Such a comparison will in turn contribute to understanding the evolution of sex pheromone production and reception in butterflies.

## Introduction

Although studies on sexual communication are biased towards visually-based traits, chemical communication using sex pheromones is predominant from arthropods to mammals for ensuring mate choice and reproduction (e.g. [1]–[4]). Sex pheromones are species-specific blends of chemical compounds used for intraspecific communication [1]. Research on several species of moths has provided case studies of the ecology and evolution of sex pheromones that are produced by females to attract mating partners, often over long distances [1], [5], [6]. Many of the active chemical components have been identified [7]–[9] and the genetics underlying pathways of pheromone production have been investigated [10]–[13]. Variation in the pheromone blend in several species has been demonstrated to be a major determinant of reproductive isolation and speciation (e.g. [5], [6], [10], [14]–[16]).

In addition, males of some species of moths and of butterflies also produce sex pheromones but their study has received much less attention and has not led to any fully comprehensive chemical and behavioural case studies. Behavioural and chemical analyses of such male sex pheromones (MSP) have been made for several species of moth [17]–[27], but to date, the MSP of only four species of butterfly have been partially analysed, namely *Pieris napi* [28], *Colias eurytheme* [29]–[33], *Danaus gilippus* [34] and *Idea leuconoe* [35]. MSP are usually employed at short–range during the courtship sequence [36]–[38] and are associated with scent-releasing organs called coremata or androconia found on the legs, wings, thorax or abdomen [37]. Male olfactory displays in Lepidoptera are thought to be involved in mate assessment as they can convey information about the prospective mates [39], [40], such as quality and quantity of nuptial gifts [21], or male size [25] (but see [41]). The diversity of both the chemical compounds and the scent-structures associated with male pheromones [7], [37] could reflect differences across the sexes in terms of patterns of recruitment of pathways at the genetical and developmental levels [42]. Additionally, a detailed comparative analysis of the biosynthesis, chemical structure, use and function of sex pheromones between male butterflies and female moths could reveal the extent of conservation versus evolutionary novelties for the behaviours, biosynthetic pathways, compounds, scent-structures and receiving systems involved in communication through sex pheromones. The development of genomics tools for several different model species of Lepidoptera [43], together with the increasing availability of robust molecular phylogenies (e.g. [44]) make such comparisons using a powerful multidisciplinary approach both more feasible and timely.

**Figure 1:**
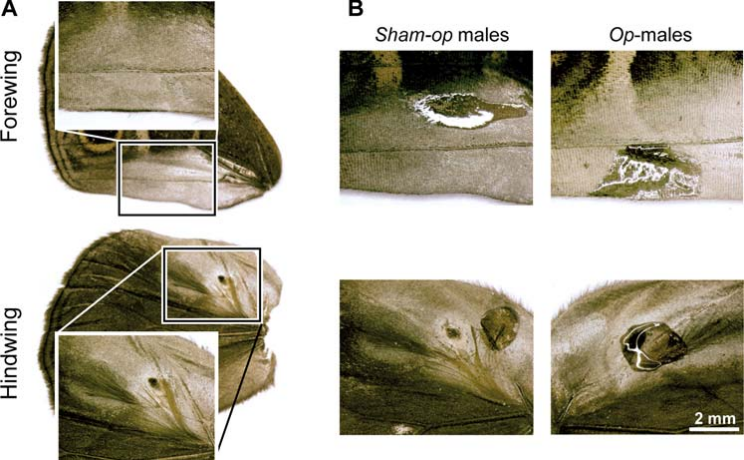
Androconia of *B. anynana* and methodology of operation of androconia. A. In *B. anynana*, the first androconia consist of a black spot of differentiated scales and of patch of yellow hairs, on the hindwing. The second androconia are formed by a silver spot of scales on the forewing and of a patch of dark hairs on the hindwing. B. Methodology of operation of androconia: after anaesthesia with CO_2_ for 15 seconds, males were mounted on an entomological wood support using pins and entomological papers to expose the androconia without damaging the wing surface. The images are representative of the result of the operation in *sham-op* (left) and *op*-males (right).

*Bicyclus* Kirby, 1871 (Lepidoptera, Nymphalidae, Satyrinae) is a species rich genus with about 80 species in sub-Saharan Africa [45]. The key taxonomic trait used by Condamin (1973) in his classic monograph of the genus were the male androconia on the wings [46]. These differentiated scales are thought to distribute scents over the female antenna during courtship [40]; they vary in number, position and morphology among species. In *Bicyclus anynana* (Butler, 1879), a species established in the laboratory since the early 1990's [43], [47]–[51], males display two pairs of androconia on the dorsal wing surfaces [46] that are each made up of modified epidermal scale cells that form patches of shiny scales, the androconial spots and hairs [37] (Fig. 1A). The first androconial structure consists of a black spot of differentiated cells between the sub-costal and radial veins on the hindwing, and a plume of yellow hairs on the hindwing cell. The second androconia are formed by a silver spot of differentiated cells on the anal vein on the forewing, and dark brown hairs between the radial and first median veins of the hindwing. Each group of hairs clearly overlies its respective androconial spot when the wings are in a resting position.

Costanzo and Monteiro (2007) [40] recently showed using a laboratory set-up that the androconia are important to *B. anynana* male mating success. Males for which the putative pheromone production of the fore- and hindwing androconial structures was blocked mated less often than control males in male-male competition experiments. They found that the chemicals emitted by both androconia were equally important for female choice. Moreover, they showed that the chemicals are likely to be perceived by the female antenna as when the latter were blocked, females were no longer able to discriminate between scented and unscented males [40].

In the present study, we first identified the male chemical components eliciting an antennal response in females using gas chromatography coupled to electro-antennographic detection (GC-EAD), and then characterized their chemical structure by gas chromatography coupled to mass spectrometry (GC-MS). We also showed that the androconial structures are involved in their production. Second, we described the courtship sequence of *B. anynana* using high speed video camera images and kinematic diagrams, and found that females usually accept or reject courting males after the pheromone transfer from the male to the female has presumably taken place. We then assessed the behavioural activity as MSP of these components in field-like conditions. Finally, we were able to restore male mating success by applying the synthetic pheromones on to the wings of operated males.

This study provides an integrative analysis of a MSP in a butterfly. The toolkit it has developed will enable the investigation of the type of information about male quality that is conveyed by the MSP. Moreover, we aim to show that *B. anynana* is relevant to contribute to the comparison of pheromone biosynthesis and function between male butterflies and female moths and, as such, improve our understanding of the evolution of sex pheromone communication in butterflies.

## Results

### 1. Characterization of the MSP

#### Identification of MSP composition

**Figure 2:**
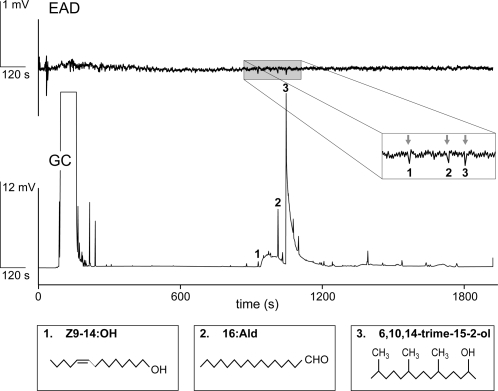
Typical GC-EAD recording showing *B. anynana* 3-day old female antennal response to a mixture of wing extracts of 3 to 8-day old males. The components 1, 2, and 3 repeatedly (n = 6) elicited a response. The chemical structures of the three active components are represented at the bottom: 1) Z9-14:OH; 2) 16:Ald; 3) 6,10,14-trime-15-2-ol.

Three components in male wing extracts were found to be repeatedly electrophysiologically active when tested using gas chromatography with electro-antennographic detection (GC-EAD) on antenna of stock females: (*Z*)-9-tetradecenol (Z9-14:OH), hexadecanal (16:Ald ) and 6,10,14-trimethylpentadecan-2-ol (6,10,14-trime-15-2-ol) (Fig. 2). The three components are sex-specific as they are not found in females. A trial in which the MSP components were compared for 60 males reared on the non-native *Zea mays* and 60 males on the natural host plant, *Oplismenus africanus*, showed no effect of larval food source on MSP titres or presence/absence of chromatogram peaks (data not shown).

**Figure 3:**
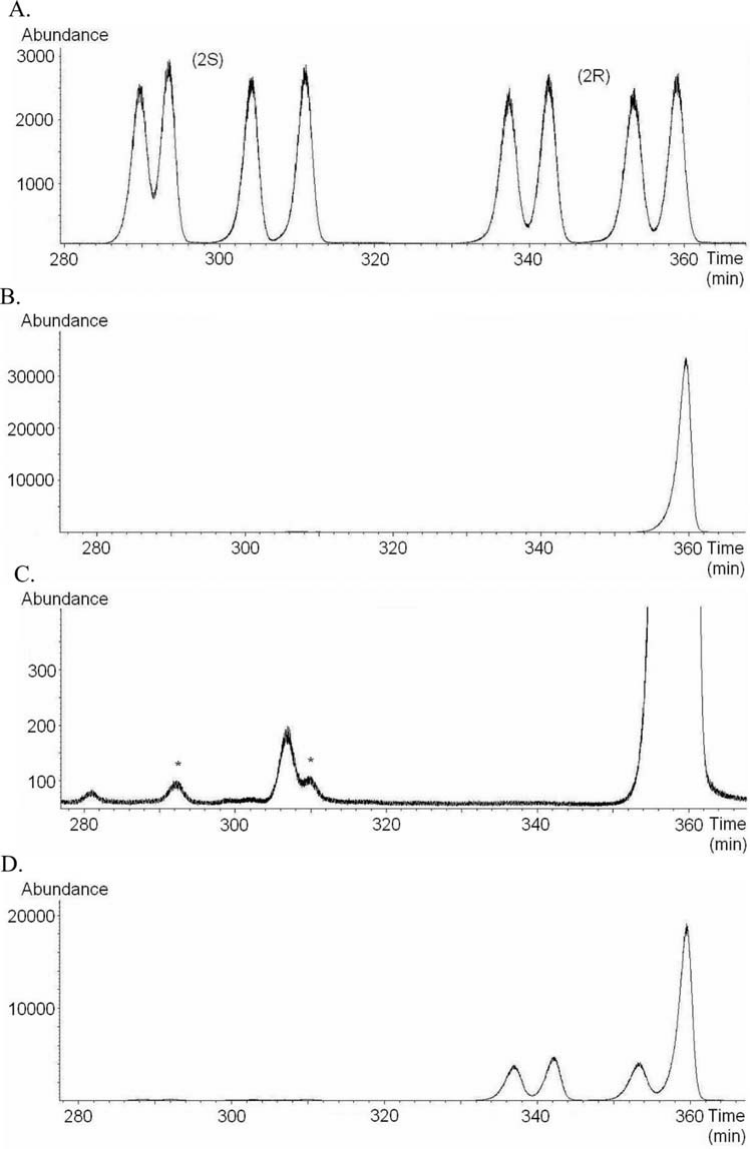
GC-MS chromatograms of derived synthetic mixtures and male wing extract samples of *B. anynana*. A. Synthetic mixture of all eight stereoisomers of 6,10,14-trime-15-2-ol (1). B. Extract of *B. anynana* with one large peak corresponding to the last peak of the synthetic (2*R*,6*R*/*S*,10*R*/*S*)- 6,10,14-trime-15-2-ol mixture (2*R*-1). C. Enlargement of the chromatogram in Figure B. Peaks marked with * have retention times corresponding to peak 2 and 4 of the synthetic (2*S*,6*R*/*S*,10*R*/*S*)- 6,10,14-trime-15-2-ol mixture (2*S*-1). Thus, the stereoisomers present in *B. anynana* males are most likely to be two of the 4 stereoisomers in the (2*S*,6*R*/*S*,10*R*/*S*)-6,10,14-trime-15-2-ol mixture (2*S*-1) as the peaks marked with * coincide with two peaks in this synthetic mixture (cf. chromatogram A). D. Wing extract of *B. anynana* spiked with synthetic (2*R*,6*R*/*S*,10*R*/*S*)-6,10,14-trime-15-2-ol mixture (2*R*-1), confirming that the peak in the extract corresponds to the final peak of the (2*R*,6*R*/*S*,10*R*/*S*)- 6,10,14-trime-15-2-ol mixture (2*R*-1).

To identify the isomeric configuration of 6,10,14-trime-15-2-ol, the retention time of the components in the extract of 200-male wings was compared with those of a derived synthetic mixture containing all eight isomers 6,10,14-trime-15-2-ol (Fig. 3A). The sample was also spiked with a synthetic mixture of (2*R*,6*R*/*S*,10*R*/*S*)-6,10,14-trime-15-2-ol (Fig. 3D). This revealed that *B. anynana* males mainly produce one stereoisomer of the 2*R*-1 mixture (99.6% of the 6,10,14-trime-15-2-ol, Fig. 3B) although the male wing extract also contains small amounts of two other stereoisomers of the 2*S*-1 mixture (Fig. 3C).

#### Location of MSP production

**Figure 4:**
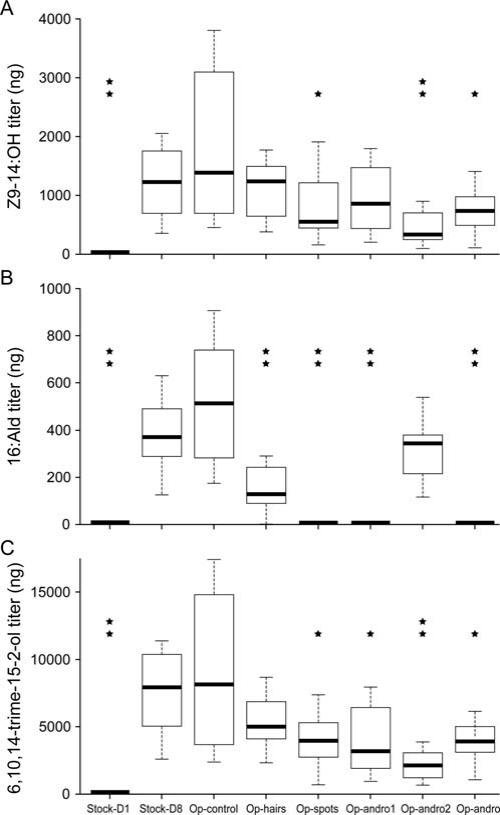
Component titres, after log-transformation, according to treatment. (A) Z9-14:OH (one-way ANOVA: R^2^ = 0.93; F_7,55_ = 98.39, *p*<2. 10^−16^); (B)16:Ald (one-way ANOVA: R^2^ = 0.94; F_7,55_ = 98.39, *p*<2. 10^−16^); (C) 6,10,14-trime-15-2-ol (one-way ANOVA: R^2^ = 0.86; F_7,55_ = 98.39, *p*<2.10^−16^). The horizontal line in each box shows the median titre for each treatment. The bottom and top of the box show the 25 and 75 percentiles, respectively. The horizontal line joined to the box by the dashed line shows 1.5 times the inter-quartile range of the data. The stars represent a significant decrease in pheromone titre compared to the *op-control* group (*: 0.01<p<0.05; **: p<0.001). Treatment codes are given in the section “Material and Methods”.

The three components of the presumptive MSP are below detection level in 1-day old stock males (*stock-D1*) and show significantly lower titres than in 8-day old stock males (*stock-D8*) (one-way ANOVA, d.f. = 7, *p*<2.10^−16^), indicating that their biosynthesis begins after adult emergence (Fig. 4). Detailed analyses reveal their site of production. The site of production of the presumptive MSP components was examined by surgical removal of different portions of the wings (n = 8 males per treatment) using fine scissors in 1-day old stock males followed by GC analysis of wing extracts made when these males were 8-day old. *Op-control* (standing for “*Operation control*”) males in which a wing area not containing the androconia was removed displayed pheromone titres equivalent to *stock-D8* males (*p*>0.37 for the three components). In contrast, removing the androconial areas significantly reduced the MSP production: Z9-14:OH titre is significantly reduced in males lacking the second androconia (*op- andro2* and *op-andro* treatments; *p*<0.02) compared to *op-control* males (Fig. 4A). More specifically, the spots (*op-spots* treatment; *p* = 0.03), but not the hairs (*op-hairs* treatment, *p* = 0.32), of the second androconia are responsible for the production of Z9-14:OH. Similar results were obtained for 6,10,14-trime-15-2-ol (Fig. 4C), except that the first androconia seem to be partly involved in its production as well (*op-andro1* treatment, *p* = 0.01). The production of 16:Ald is completely suppressed in males lacking the first androconia (*op- andro1* and *op-andro* treatments; *p*<2.10^−16^), and production of 16:Ald relies on both patches of hairs (*op-hairs* treatment; *p*<2.10^−5^) and spots (*op-spots* treatment; *p*<2.10^−16^) (Fig. 4B).

### 2. Role of the presumptive MSP in male mating success

#### Courtship description

**Figure 5:**
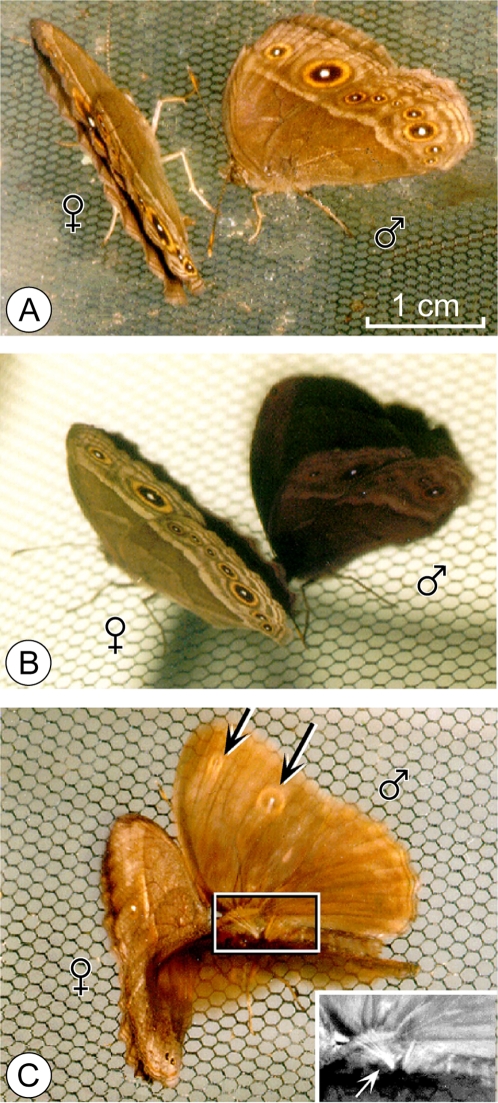
Courtship sequence of *B. anynana*. Steps 2 to 4 are represented here: A. Orientation: the male assumes a position at approximately 45° relative to the female body axis. B. Flickering: the male moves his wings jerkily and speedily, while the female stands. C. Thrust: the male opens his wings, revealing the fan-like hairs on the dorsal surface of the hindwing (within box with enlargement to lower right). Pictures from [52].

Six consecutive steps were detected in a typical successful courtship (Fig. 5) [52]. The male first locates and approaches a female (*localization*). Second, the male orientates its body towards the posterior part of the female (*orientation*); both butterflies have their wings closed. The angle formed between the bodies of the two butterflies ranges from 45° to 90°. Third, the male initiates a rapid sequence of opening and closing its wings (*flickering*; ca 5–17 flicks per second). High speed video images reveal that the wings open and close with a rolling movement during individual flicks, and that during bouts of flickering, the average angle of wing opening increases while the flickering pace decreases (about 5 flicks per second). Fourth, the *thrust* phase during which the male touches the edge of the female wings with his head; his antenna or legs may also make contact with the side of the female. Simultaneously, the wing tips eventually open completely so as to touch the substrate. During the *thrust*, the two sets of androconial hairs fan out above the wing surface and become clearly visible when the wings are open (see arrow in Fig. 5C). Fifth, the male curls his abdomen in order to grasp the tip of the female abdomen with his claspers (*attempting*). Following a successful genital contact, the male moves until the copula position typical of Lepidoptera is reached (*copulation*). The average courtship duration is only a few seconds (14.3±2.38 s; mean±SE) whilst the average copulation lasts about 30 minutes [53]. The spatial orientation between the male and female during courtship is mainly determined by changes in the position of the male. The female usually stands still regardless of male behaviour except when she rejects the male (see below).

**Figure 6:**
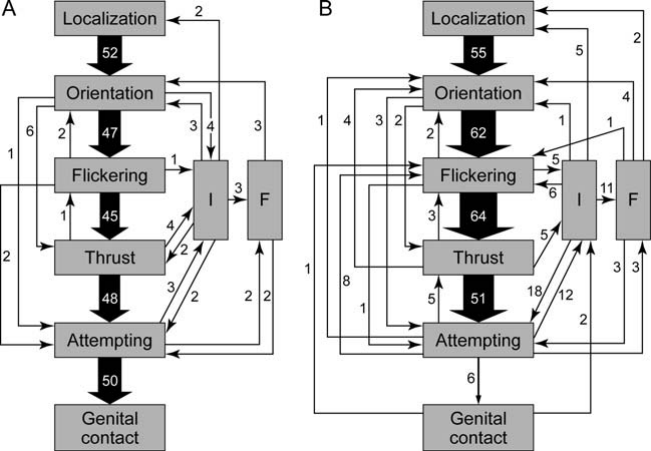
Kinetic diagram of courtship behaviour based on 50 successful (A) and 50 unsuccessful (B) courtships. For each behavioural step (in boxes), the frequency of transitions to other courtship steps is indicated by an arrow and by adjacent figures giving the number of each type of transition (from [52]). Interruptions of the general behavioural sequence are noted by “I”. “Following” of a female by the male during courtship, noted as “F”, involved in 80% of the cases short flights. In (B), courtship stopped at the flickering (6 cases), thrust (9 cases), attempting (32 cases), genital contact (2 cases) or “F” (1 case) steps.

The comparison of 50 successful and 50 unsuccessful courtship sequences using kinetic diagrams (Fig. 6) revealed that females are most likely to reject males directly after the thrust phase (Chi-Square test: χ^2^, 0.05 = 48.51, d.f. = 5, *p*<0.001) [52]. Female rejection behaviour consists of walking or flying away, and it interrupts (step “I” in Fig. 6) more often the male courtship sequence in unsuccessful than in successful sequences (χ^2^, 0.05 = 51.32, d.f. = 1, *p*<0.001) suggesting that females choose to accept or reject on the basis of outcome of the male courtship display. In about a third of the cases, the male followed (step “F” in Fig. 6) the female after courtship interruption in order to resume courtship. Moreover, successful courtships tend to be shorter (Mann-Whitney test: n = 50, W = 792, *p* = 0.07; mean±SE for successful courtships is 14.3±2.38 s; mean±SE for unsuccessful courtships is 22.20±3.45 s), and less complex than unsuccessful ones: the sequence of six typical steps is more likely to be interrupted in unsuccessful courtships (Mann-Whitney Test, n = 50, W = 1984, *p* = 0.0001).

#### Mating success of males with and without MSP

**Figure 7:**
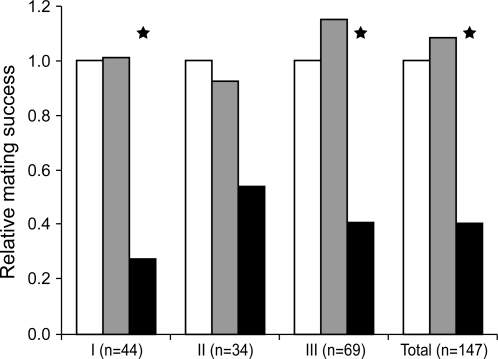
Mating success of *sham-op* (grey bars) and *op*-males (black bars) relative to that of stock males (white bars). Three trials (I to III) were performed with butterflies drawn at random from the stock population. Stock male mating success is set at 1. The total number of matings (n) is given for each trial. G-test significance: asterisk, *p*<0.05.

Competition experiments were performed in semi-natural conditions to test the effect of the MSP in mate choice. We allowed free flight and a full expression of courtship behaviour in these experiments which resulted in a high mating rate of females (62%) while ensuring a relatively high competition for pairings between males. We released groups of male butterflies with the androconial structures absent (*op*-males) or present (*sham-op* and stock males). There was evidence of differential male mating success in two of the three trials (I-II-III; G-test for the stock : *sham-op* : *op* comparison, Fig. 7), and there was no heterogeneity among trials (Pearson's Chi-squared test; *p*>0.45 for each comparison; Table 1). The difference in mating success for the pooled data lies between *op-* and both *sham-op* and stock males (G-test for the *op* : (*sham-op*+stock) comparison, G = 72.17, d.f. = 1, *p* = 10^−16^), and not between stock and *sham-op* males (G-test for the stock : *sham-op* comparison, G = 0.008, d.f. = 1, *p* = 0.93). *Op*-males achieved only 15% of all matings, representing a mating success of about one-third that of either stock or *sham-op* males (which achieved 43 and 42% of all matings, respectively). Males of the three groups were captured in similar numbers at the end of each experiment indicating similar survivorship (I-II-III; G-test for the stock : *sham-op* : *op* comparison). Since application of varnish to treat the males significantly decreases the titres of the three pheromone components from a third to a sixth of the usual titre levels (n = 30, Mann-Whitney test, n = 30, *p*<0.001), the lower mating success of operated males is likely to be due to the low level of the components released by the androconia.

**Table 1:** Female mating and male recapture in greenhouse experiments for stock, *sham-op* and operated groups.

Replicates	I	II	III	Total I-II-III
	Stock∶Sham-op∶Op	Stock∶Sham-op∶Op	Stock∶Sham-op∶Op	Stock∶Sham-op∶Op
Dust color	Y∶R∶B	B∶Y∶R	R∶B∶Y	
**Females**				
Released	90	120	90	300
Recaptured	65	47	68	180
Recaptured (%)	72.2	39.2	75.5	62.3
Mated with				
Stock	19	17	27	63
Sham-op	20	11	31	62
Operated	5	6	11	22
Stock/Sham-op	2	0	6	8
Stock/Operated	0	1	2	3
Sham-op/Operated	0	0	1	1
Unmated	21	13	5	39
G-test Stock∶Sham-op∶Op	G,2df = 9.59, *p* = 0.008	G,2df = 5.35, *p* = 0.069	G,2df = 9.74, *p* = 0.008	G,2df = 22.32, *p*<10−4
**Males**				
Released	40∶40∶40	58∶58∶58	40∶40∶40	138∶138∶138
Recaptured	72	54	63	189
Recaptured (%)	60	31	52.5	47.8
Stock	24	23	21	68
Sham-op	25	16	21	62
Operated	23	15	21	59
G-test Stock∶Sham-op∶Op	G,2df = 0.08, *p* = 0.96	G,2df = 2.11, *p* = 0.35	G,2df = 0.00, *p* = 1.00	G,2df = 0.67, *p* = 0.72

For males, the figures are the absolute numbers after recapture for each male group. For females, the figures are the numbers of recaptured females that mated with each male group. Dust colors were blue (B), red (R) and yellow (Y). Overall recapture rates (%) are given for each experiment.

#### Recovery of mating success of males perfumed with MSP synthetics

To demonstrate that the identified compounds constitute the effective MSP of *B. anynana* we attempted to restore the mating success of *op*-males by adding the synthetic components onto their wing surfaces. The measurement of evaporation rates of the synthetic components when a 10-male equivalent load was applied onto the wings of female butterflies showed that 50 percent of the extract is lost immediately after application. Changes of ratios between components became biologically significant after 2.5 to 3 hours (Tukey's HSD correction for multiple comparisons, *p* = 0.001).

We released groups of 8-day old *op*-males, scented with a 10-male equivalents of the synthetic MSP component candidates (*perfumed* males) or not (*control* males). A series of pilot competition trials under various conditions showed that ambient temperature (optimal range between 26–29°C) and female age (3 to 6-day old) were important in determining male mating success. When using the 2-component blend (16:Ald+Z9-14:OH), females mated significantly more with *perfumed* males when using the 2-component extract than with *control* males (I-II, Table 2; G = 5.41 and 4.57 respectively, d.f. = 1, *p*<0.03). There was no heterogeneity among trials (Pearson's Chi-squared test; *p*>0.7), and the pooled data sets confirmed a significantly higher mating success of *perfumed* males (G = 9.96, d.f. = 1, *p*<0.002). *Control* males achieved only 20% of all matings. This effect was not observed when the 3-component extract was used (III–IV, Table 2): pooled data sets (Pearson's Chi-squared test; *p*>0.95) showed that *perfumed* and *control* males displayed similar mating success (G = 0.13, d.f. = 1, *p* = 0.71).

**Table 2:** Female mating in competition experiments for *perfumed* and *control* male groups.

Replicates	Z9-14:OH+16:Ald		Z9-14:OH+16:Ald+6,10,14-trime-15-2-ol	
	I	II	III	IV
**Females mated with**				
Perfumed	12	11	8	8
Control	3	3	8	6
Perfumed+control	0	0	0	0
Unmated	5	6	4	6
**G-test Perfumed+Control**	G,1df = 9.96, *p* = 0.002		G,1df = 0.13, *p* = 0.71	

In the first two trials, the extract contained only Z9-14:OH and 16:Ald, while in the last two trials, the extract of perfumed males consisted of the three synthetic components. The figures are the numbers of recaptured females that mated with each male group. Dust colors were red (R) and yellow (Y).

## Discussion

We have identified three components included in the sex pheromone of *B. anynana* males, namely Z9-14:OH, 16:Ald and 6,10,14-trime-15-2-ol. The effect of the latter component was shown by GC-EAD and in a competition experiment between *op-*, *sham-op* and stock males but, unlike the former two, we could not restore the mating success of *op*-males when perfumed with the synthetics including the 2*R*-1 mixture. Thus the pheromonal activity of 6,10,14-trime-15-2-ol is not conclusively demonstrated. The behavioural activity of the most abundant isomer of 6,10,14-trime-15-2-ol may have been counteracted by the presence of additional stereoisomer(s) in the 2*R*-1 mixture that acted as behavioural antagonist(s) (inhibitory signal) [39], [54], [55], and thus cancelling out the positive effect of Z9-14:OH and 16:Ald. Moreover, we cannot exclude that the two minor isomers (<0.04% of the total MSP) identified in the butterfly wing extract and part of the 2*S*-1 mixture may also contribute to the MSP. As antagonistic isomers can be involved in reproductive isolation of closely related moth species [7], and 6,10,14-trime-15-2-ol has generated GC-EAD responses in other *Bicyclus* species (Nieberding, unpubl. data), the additional synthesis of the different individual isomers might be necessary for future pheromone analysis across the *Bicyclus* genus.

This study provides an integrative analysis of the MSP blend in males of a butterfly species. Behavioural and chemical analyses of MSP have been made for several species of moth and has also been analysed in part for four species of butterfly (see Introduction), but these species do not have a toolkit comparable to that available in *B. anynana* [43]. We have shown that the transfer of pheromone from males to females in *B. anynana* is probably associated with the flickering and thrust phases of courtship. We also identified by GC-EAD the male components that induced a female antennal response, characterized their chemical structure, and showed that the androconia are involved in their production which occurs after adult emergence. Moreover, we assessed the behavioural activity of two of these components in semi-natural conditions. The necessity for validations of behavioural data in semi-natural conditions has been highlighted recently in *B. anynana* as the conditions of captivity may influence behavioural responses and affect the expression of key courtship traits [56]. Taken together, the absence of supplementary secondary sexual structures on the body of males [46], the absence of novel synthesized components by males reared on the native food diet, and the recovery of male mating success of *perfumed op*-males, suggest that the three identified components form the MSP of *B. anynana*.

### The role(s) of sex pheromones in male mating success

*B. anynana* MSP is effective at close-range courtship, similarly to MSP identified in moths, but in contrast to the long-range attractiveness of female moth blends. Pheromones released by females in many species of moths are known to be important in species and sex recognition. A few studies have specifically tested the activity of sex pheromones independently for these functions [57]. In *B. anynana*, we have demonstrated the importance of MSP in mating success, but the experiments could not distinguish what specific type of information MSP convey. Costanzo and Monteiro [40] suggested that the MSP in *B. anynana* could be involved in male to male recognition as they observed that males would often attempt to court males that had their androconial structures blocked. Additionally, sexual selection by females could be the main driving force of *Bicyclus* pheromone evolution, and be a crucial component in the process of speciation [40], in a similar way as has been recently suggested in female moths (e.g. [5]). Indeed, MSP titres and ratios display a high variability in stock males of *B. anynana* (data not shown) providing an opportunity for sexual selection. Moreover, our analysis of courtship revealed that virgin females frequently reject one or more males before accepting a mate, and that female rejection usually occurs directly after the flickering and thrust phases when MSP transfer probably occurs suggesting that females could use MSP to assess the quality of individual males [40]. If sexual selection is directly involved in male mating success, variation in MSP blend is expected to be associated with variation in male “quality”. The toolkit developed in this study for *B. anynana* (e.g. methodology of micro-surgical manipulations, synthetic pheromone components and experimental competition assays) will enable the relevant experiments to be performed to investigate the type of information that is conveyed by *B. anynana* MSP. In addition, we will make use of a wing eyespot mutant we have established in the lab (*comet* [48]) which has reduced androconial structures and lacks one of the MSP components (Nieberding, unpubl. data).

#### Exploring the evolution of sex pheromone biosynthesis in Lepidoptera

Two components of *B. anynana* MSP, namely 16:Ald and Z9-14:OH, have been identified as components of the female sex pheromone (FSP) in numerous species of moth, the so-called “type I” pheromones [7], [8], [58] (for an exhaustive list, consult the pherobase, http://www.pherobase.com/). Male pheromone components similar to the fatty-acid derivatives Z9-14:OH and 16:Ald were also recently identified in the green-veined Pierid butterfly, *Pieris napi* [28], while the use of 6,10,14-trime-15-2-ol as a MSP remains, to our knowledge, a unique observation in butterfly species. “Type I” moth sex pheromones are produced from fatty acids through the sequential activity of a few enzymes involved in fatty acid metabolism and conserved over the tree of life: some of which shorten the carbon chain length (ß-oxidase), insert double bonds (desaturase) or add functional groups (oxidase, reductase) [7], [8]. Beyond this level of conservation, moths are believed to have a series of unique desaturases that produce the variety of unsaturated compounds exhibited in their sex pheromone blends [8], [59]. Unique desaturases include the delta(Δ)11 desaturase which has only been found in moths. Some moth species make a use of the Δ11 desaturase, and not of the ubiquitous Δ9 desaturase common to all animals, to produce Δ9–14 compounds from Δ11–16:acids via a step of chain-shortening [59], [60].

In this respect, our finding that males of the butterfly *B. anynana* produce pheromone components identical to those used by female moths, including the Z9-14:OH component, provides for the first time to our knowledge an experimental system that could be used to examine the extent of similarity of pheromone biosynthesis between butterflies and moths. Indeed, the synthesis of Z9-14:OH in *B. anynana* males can involve either the Δ9desaturase or the moth-specific Δ11desaturase. This alternative can be tested in *B. anynana* using the genetic and genomic tools available for the species. It will involve the searching and characterization of putative Δ11desaturase genes from our butterfly genomic database [61] and the realization of a functional assay to show whether this gene introduces a double bond at 11^th^ or any other position. Alternatively, the use of labeled precursors may help adress this question (as e.g. in [60]).

Similarity of biosynthesis can either be due to identity by descent –pathways in butterflies were conserved from their moth ancestors [44], [62], [63]-, or can be due to convergence, in which case these would represent evolutionary pathways of least resistance. In any case, information from additional butterfly lineages, including more primitive, non-satyrine species, and from moth species phylogenetically close to the butterflies, will be necessary to investigate the extent of similarity in pheromone biosynthesis in a phylogenetically relevant context.

Finally, the receiving systems of pheromone-binding proteins and receptors that form a lock-and-key system for pheromone components in male moth antenna [64] are also likely to display similar structures to those in female butterflies. Thus, degree of similarity in the enzymatic and developmental pathways of both the production and reception of pheromones between female moths and male butterflies could be investigated. Together, our results open up for a detailed comparative analysis for pheromone communication between male butterflies and female moths, and they highlight the potential value of the *Bicyclus* system for contributing to the understanding of the evolutionary patterns and processes involving the sex pheromones in butterflies.

## Materials and Methods

### Insects

An outbred laboratory stock of the African butterfly, *B. anynana*, was established in 1988 from over 80 gravid females collected in a single source population in Malawi. It is maintained on a maize-based diet. In order to preserve high levels of heterozygosity population size ranges between 400 to 600 adults per generation [53]. Adult butterflies are fed on mashed banana *Musa acuminata*. Unless stated otherwise, the experiments were performed on stock individuals reared in climate rooms under a standard temperature regime (27.5±0.5°C) and high relative humidity representing the wet season in Malawi.

### Chemicals

**Figure 8:**
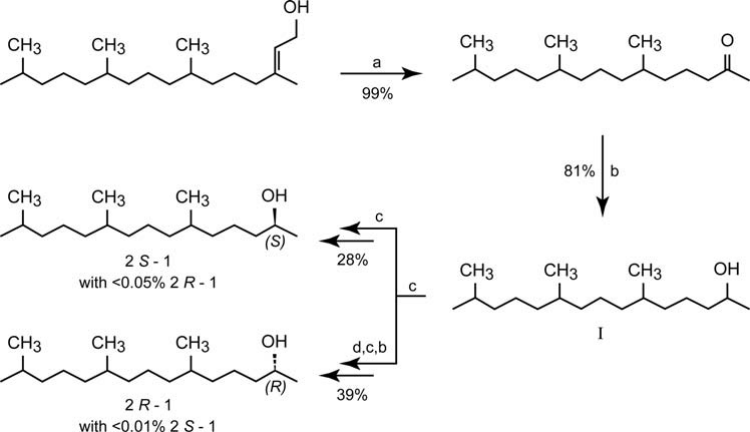
Synthesis of a mixture of all eight stereoisomers of 6,10,14-trime-15-2-ol (1), of (2*R*,6*R*/*S*,10*R*/*S*)-6,10,14-trime-15-2-ol (2*R*-1) and of (2*S*,6*R*/*S*,10*R*/*S*)-6,10,14-trime-15-2-ol (2*S*-1). (a) NaIO_4_, RuCl_3_, CH_3_CN, H_2_O. (b) (i) LiAlH_4_/Et_2_O. (ii) H_2_O/H_3_O^+^. (c) (i) CALB, vinyl acetate, heptane, 4Å molecular sieves. (ii) LC-separation of the produced 2*R*-acetate and the remaining 2*S*-alcohol. (d) Hydrolysis of the 2*R*-acetate using KOH/MeOH (2,4M).

(*Z*)-9-tetradecenol (Z9-14:OH) is available from Sigma-Aldrich. Hexadecanal (16:Ald) was synthesized from hexadecanol (Fluka) via a Dess-Martin oxidation and was obtained in 91% yield and with 99.6% chemical purity [65] . The synthesis of the stereoisomeric mixtures of 6,10,14-trimethyl-pentadecan-2-ol (1) (6,10,14-trime-15-2-ol; Fig. 8), as based in parts on published procedures, started from phytol (Sigma-Aldrich) which was nearly quantitatively oxidized at room temperature by sodium periodate and a catalytic amount of ruthenium (III) chloride [26]. The obtained product, ketone, was purified to a chemical purity of 95% by liquid chromatography and then reduced by LiAlH_4_ in 81% yield to give a mixture in equal amounts of the eight possible stereoisomers of 6,10,14-trime-15-2-ol with a chemical purity of 99%. We have previously successfully used lipase catalysed acylation of similar alcohols to obtain 2*S*-isomers pure from 2*R*-isomers and vice versa [66], [67]. Thus, we applied this methodology on the 6,10,14-trime-15-2-ol, using vinyl acetate as acyl donor and *Candida antarctica* lipase-B (CALB) as catalyst under dry conditions. We obtained after LC-separation the remaining alcohol 2*S*-1 (with <1% of 2*R*-1 stereoisomers and a chemical purity of >99%) in 49% yield and 2*R*-1 acetate, which after hydrolysis furnished 2*R*-1 (with <1% of 2*S*-1 stereoisomers and a chemical purity of >99%) in 35% yield. Repeating the lipase catalyzed acylation on both the stereoisomerically enriched alcohols 2*S*-1 and 2*R*-1, with a conversion of 20% and 79% respectively, we obtained after LC-separation and hydrolysis of the 2*R*-1 acetate both alcohols in high purity (77% yield of 2*R*-1 with <0.01% of 2*S*-1 stereoisomers and a chemical purity of >99% and 80% yield of 2*S*-1 with <0.05% of 2*R*-1 stereoisomers and a chemical purity of >99%). The full experimental information is provided in the following supporting information file Text S1: Synthesis of 16:Ald and of the stereoisomeric mixtures of 6,10,14-trime-15-2-ol.

### Characterization of the presumptive MSP

#### Identification of MSP composition

Volatile components containing the candidate sex pheromone were extracted by soaking the wings of five males for 15 minutes in 1 ml of hexane. The extracts were analyzed on a Hewlett-Packard 6890 series II gas chromatograph (GC) equipped with flame-ionization detector and interfaced with a HP- 6890 series integrator. The carrier gas was nitrogen. The injector temperature was set at 240°C and the detector temperature at 250°C. A HP-1 column was used and the oven temperature was increased from the initial temperature of 50°C by 15°C/min up to a final temperature of 295°C maintained for 6 min.

To identify the male pheromone components, a HP-5890 series II GC equipped with a HP-1 column was used. Male wing extracts were injected splitless and the injector temperature was set at 240°C. Hydrogen was used as carrier gas and the temperature of the column was kept at 50°C for 2 min, increased by 10°C/min up to 250°C as final temperature kept for 2 min. The injected sample was split between two outlets allowing simultaneous recording of the chromatographic pattern (GC) and of the electrophysiological response of female antenna (EAD). Nitrogen was used as make-up gas for the split. Antenna of 3 to 5-day old females were cut off at the first basal segment and placed between the two electrodes of an EAG probe (Syntech, The Netherlands) using electrically conductive gel. All GC-EAD recordings were analyzed with Autospike 32 software (Syntech, The Netherlands).

The identification of the compounds that repeatedly elicited antennal responses (named hereafter presumptive MSP components) was performed by analyzing male wing extracts with a HP-5972 mass spectrometer (GC-MS) in scan mode. The compounds were identified by comparison of their spectra with standard mass spectra and their retention times with the corresponding synthetic references. The isomeric configuration of one of the three presumptive MSP components, Z9-14:OH, was identified using dimethyl disulfide derivatization of wing and synthetic [68]. The formed adducts were analyzed by GC-MS. The identification of the isomeric configuration of a second presumptive MSP component, 6,10,14-trime-15-2-ol, required making an extract using 200-male-wings that was derived using chrysanthemoyl chloride [69]. The diastereomers formed in this way were analysed with a Hewlett-Packard 6890N GC fitted with a polar factor FOUR VF-23ms column (Varian, 30 m×0.25 mm i.d.) and a HP 5973 mass spectrometer (GC-MS) in SIM mode (m/z = 123). The carrier gas was helium; 1 µl of the sample was injected splitless, the injector temperature was 250°C and the transfer line temperature was 280°C. The column temperature was increased from 50°C by 10°C/min up to 110°C, from 110°C by 0.02°C/min up to 125°C, and from 125°C by 10°C/min up to 200°C.

#### Location of MSP production

MSP titres of 1-day old (*stock-D1*) and 8-day old (*stock-D8*) stock males were measured in non-manipulated stock males. In parallel, the site of production of the presumptive MSP components was examined by surgical removal of different portions of the wings (n = 8 males per treatment) using fine scissors in 1-day old stock males followed by GC analysis of wing extracts made when these males were 8-day old, as described previously. A wing area not containing the androconia was removed in *op-control* (standing for “*operation control*”) males. Androconial hairs were removed in *op-hairs* males, while *op-spots* males lacked the androconial spots. Males lacking the first or the second androconia were designated as *op- andro1* or *op- andro2*, respectively, while all androconial structures were removed in *op-andro* males. Hexadecyl acetate was used as internal standard to quantify the absolute amounts (in nanograms) of MSP components. Pheromone titres were first log-transformed and transformed data were analyzed by one-way ANOVA.

### Role of the presumptive MSP in male mating success

#### Courtship description

Courtship was studied in netting cages containing from 30 to 70 7-day old virgin males and females. We recorded behaviour using a GF-S1000He JVC video camera with VHS or SVHS video tapes (25 frames per second). High-Speed Video (HSV) recordings were also made (500 frames per second) to detail the courtship steps (see below). Subsets of video recordings of 50 successful and 50 unsuccessful courtships were chosen randomly for detailed analysis by projection of single frames. Additional observations were also made using free-flying butterflies in a spacious greenhouse to check that laboratory-based data were representative of natural behaviour [56].

#### Mating success of males with and without MSP

To test the role of presumptive MSP components in male mating success we performed mating competition experiments in semi-natural conditions using males with or without androconial structures. More specifically, virgin stock males were randomly assigned at emergence to one group: control, operated (*op*), or sham-operated (*sham-op*). *Op-* and *sham-op* males were prepared by applying a transparent nail solution (Revlon Liquid Quick Dry; see [40]) onto the wings at 1-2 days of age. This solution is an efficient scent-blocking solution in *B. anynana* [40]. *Op*-males received the solution on the androconial spots while the hairs were removed using fine scissors (Fig. 1B). In *sham-op* males, the solution was applied adjacent to the androconial spots and the hairs were brushed but not cut. These treatments maintained flight ability comparable to control males. We then dusted the genitalia of the males with colored ‘rodent-tracking’ fluorescent dust using a different color for each competing group; the dust is transferred to the female partner's genitalia during mating [56]. Colors were switched across groups between experiments.

Forty to sixty 2 to 7-day old virgin males were released in a spacious tropical greenhouse that provided a semi-natural environment for *B. anynana* (cf. [56]). Density was comparable to field populations [70]. On the following morning, 2 to 5-day old virgin stock females were released to reach a 2∶1 male∶female ratio. A second group of females, half the size of the first group, was released 1 day later. Equal numbers of males of differing treatments competed for matings over 48 h. All butterflies were collected 1.5 days after the final group of females was released. Females were inspected under ultraviolet illumination for fluorescent dust transferred at mating to assess the group-identity of their mating partners. Occasional double matings were scored as 0.5∶0.5. Three trials were performed sequentially. Data analysis used G-tests. In each replicate, male survival and mating distributions were compared with a 1∶1∶1 or a (1+1)∶1 distribution (stock, *sham-op* and *op-* groups). We tested for heterogeneity among replicates using a Pearson's Chi-squared test (or a Fisher's Exact test if the expected frequencies were close to 5 or less) before performing the same analyses on the pooled data set.

#### Recovery of mating success of males perfumed with MSP synthetics

Competition experiments were performed to test if the mating success of *op-*males could be increased by artificially restoring their MSP blend. We used *op-*males supplemented with the previously identified pheromone components (*perfumed* males) or not supplemented (*control* males).

As pheromone components can have differential evaporation rates, and because ratios among pheromone components may be critical for mating success [7], we determined the evaporation rates at 27°C of the synthetic components after addition on *B. anynana* wings. We took advantage of the observation that stock females produced no putative MSP components. A 10-microliter extract containing 10-male equivalent of the MSP of 8-day old males diluted in hexane (that is 20, 2.7 and 100 micrograms of Z9-14:OH, 16:Ald and the 2*R*-1 mixture of (R/S)6,(R/S)10,14-trime-15-2-ol, respectively) was applied onto the wings of each female. A 10-male equivalent load was chosen to maximize the difference in MSP between *control* and *perfumed* males and to tentatively counteract the elimination of the hairs which normally help to distribute the MSP. We used GC to quantify each component at a series of 7 times from 5 to 300 minutes after application (5 females/sample point). Multiple comparisons corrected by Tukey's honestly significant difference were used to compare ratios. The duration of the competition experiment between *control* and *perfumed* males was set at the time point when a significant difference in MSP ratios was detected.

Competition between *perfumed* and *control* males for matings with stock females was performed in a 3×3×3 m net-covered wood-framed cage placed in a greenhouse kept at 27–29°C. The cage was lit by a set of SHP sodium, HTX MetalHalide and high UVA and UVB lamps, and mirrors were placed on three sides of the cage to limit light dispersion outside the cage. This set up allowed us to mimic the range and amount of wavelengths observed in natural daylight. In addition, we could release and recapture all scented butterflies within the time limit imposed by the evaporation rate of the synthetic MSP. Groups of stock males were operated the day after emergence as described above. At 7 to 9-day old, the genitalia of 30 *op*-males were painted with fluorescent dust. The next day, they were perfumed with an extract containing 10-male equivalent of 8-day old males (15 *perfumed* males) or with a 10-microliter hexane (15 *control* males), and then immediately released together with 20 virgin 3 to 5-day old females. The composition of the extract was checked before each trial by GC analysis. Four replicates were performed sequentially: in the first two trials, the extract contained only Z9-14:OH and 16:Ald, while in the final two trials, the perfume consisted in a blend of the three synthetic components. The relative mating success of *perfumed* versus *control* males was determined as above.

### Statistical analyses

All statistical analyses were carried out using the R language and environment for statistical computing and graphics (http://www.r-project.org/). Pheromone titres were log-transformed, and the ratios between pheromone components were cube-root-transformed [71], so as to best normalize the distribution of the residuals, homogenize the variance in the data set, and maximize the fit of the model to the data set. Simplification of models was carried out using the AIC criterion which penalizes more complex models with equal fit.

## Supporting Information

Text S1 Synthesis of 16:Ald and of the stereoisomeric mixtures of 6,10,14-trimethylpentadecane-2-ol.
(0.04 MB DOC)

## References

[1] Wyatt TD (2003). Pheromones and animal behaviour..

[2] Bigiani A, Mucignat-Caretta C, Montani G, Tirindelli R (2005). Pheromone reception in mammals.. Rev Physiol Biochem Pharmacol.

[3] Ferveur JF (2005). Cuticular hydrocarbons: Their evolution and roles in *Drosophila* pheromonal communication.. Behav Genet.

[4] Symonds MRE, Elgar MA (2008). The evolution of pheromone diversity.. Trends Ecol Evol.

[5] Roelofs WL, Liu WT, Hao GX, Jiao HM, Rooney AP (2002). Evolution of moth sex pheromones via ancestral genes.. Proc Natl Acad Sci U S A.

[6] Roelofs WL, Rooney AP (2003). Molecular genetics and evolution of pheromone biosynthesis in Lepidoptera.. Proc Natl Acad Sci U S A.

[7] Ando T, Inomata S, Yamamoto M (2004). Lepidopteran sex pheromones.. Topics Current Chem.

[8] Jurenka R (2004). Insect pheromone biosynthesis.. Topics Current Chem.

[9] Mori K (2004). Pheromone synthesis.. Topics Current Chem.

[10] Löfstedt C (1993). Moth pheromone genetics and evolution.. Philos Trans R Soc Lond B Biol Sci.

[11] Groot AT, Ward C, Wang J, Pokrzywa A, O'Brien J (2004). Introgressing pheromone QTL between species: towards an evolutionary understanding of differentiation in sexual communication.. J Chem Ecol.

[12] Rafaeli A (2005). Mechanisms involved in the control of pheromone production in female moths: recent developments.. Entomol Exp Appl.

[13] Sheck AL, Groot AT, Ward CM, Gemeno C, Wang J (2006). Genetics of sex pheromone blend differences between *Heliothis virescens* and *Heliothis subflexa*: a chromosome mapping approach.. J Evol Biol.

[14] Löfstedt C, Herrebout WM, Menken SBJ (1991). Sex pheromones and their potential role in the evolution of reproductive isolation in small ermine moths (Yponomeutidae).. Chemoecology.

[15] Svensson M (1996). Sexual selection in moths: the role of chemical communication.. Biol Rev Camb Philos Soc.

[16] Groot AT, Horovitz JL, Hamilton J, Santangelo RG, Schal C (2006). Experimental evidence for interspecific directional selection on moth pheromone communication.. Proc Natl Acad Sci U S A.

[17] Baker TC, Nishida R, Roelofs WL (1981). Close-range attraction of female oriental moths to herbal scent of male hairpencils.. Science.

[18] Nishida R, Baker TC, Roelofs WL (1982). Hairpencil pheromone components of male oriental fruit moths, *Grapholita molesta* (Lepidoptera, Tortricidae).. J Chem Ecol.

[19] Phelan PL, Silk PJ, Northcott CJ, Tan SH, Baker TC (1986). Chemical identification and behavioral characterization of male wing pheromone of *Ephestia elutella* (Pyralidae).. J Chem Ecol.

[20] Teal PEA, Tumlinson JH (1989). Isolation, identification, and biosynthesis of compounds produced by male hairpencil glands of *Heliothis virescens* (F) (Lepidoptera, Noctuidae).. J Chem Ecol.

[21] Dussourd DE, Harvis CA, Meinwald J, Eisner T (1991). Pheromonal Advertisement of a Nuptial Gift by a Male Moth (*Uthetiesa ornatrix*).. Proc Natl Acad Sci U S A.

[22] Jacquin E, Nagnan P, Frerot P (1991). Identification of hairpencil secretion from male *Mamestra brassicae* (L) (Lepidoptera, Noctuidae) and electroantennogram studies.. J Chem Ecol.

[23] Heat RR, Landolt PJ, Dueben BD, Murphy RE, Schneider RE (1992). Identification of male cabbage looper sex pheromone attractive to females.. J Chem Ecol.

[24] Kimura T, Honda H (1999). Identification and possible functions of the hairpencil sent of the yellow peach moth, *Conogethes punctiferalis* (Guenee) (Lepidoptera: Pyralidae).. Appl Entomol Zool.

[25] Iyengar VK, Rossini C, Eisner T (2001). Precopulatory assessment of male quality in an arctiid moth (*Uthetiesa ornatrix*): hydroxydanaidal is the only criterion of choice.. Behav Ecol Sociobiol.

[26] Sasaerila Y, Gries R, Gries G, Khaskin G, King S (2003). Sex pheromone components of male *Tirathaba mundella* (Lepidoptera : Pyralidae).. Chemoecology.

[27] Hillier NK, Vickers NJ (2004). The role of heliothine hairpencil compounds in female *Heliothis virescens* (Lepidoptera : Noctuidae) behavior and mate acceptance.. Chem Senses.

[28] Andersson J, Borg-Karlson A-K, Vongvanich N, Wiklund C (2007). Male sex pheromone release and female mate choice in a butterfly.. J Exp Biol.

[29] Grula JW, McChesney JD, Taylor OR (1980). Aphrodisiac Pheromones of the Sulfur Butterflies *Colias eurytheme* and *Colias philodice* (Lepidoptera, Pieridae).. J Chem Ecol.

[30] Sappington TW, Taylor OR (1990). Developmental and environmental sources of pheromone variation in *Colias eurytheme* butterflies.. J Chem Ecol.

[31] Sappington TW, Taylor OR (1990). Genetic sources of pheromone variation in *Colias eurytheme* butterflies.. J Chem Ecol.

[32] Sappington TW, Taylor OR (1990). Disruptive sexual selection in *Colias eurytheme* butterflies.. Proc Natl Acad Sci U S A.

[33] Taylor OR (1973). Reproductive isolation in *Colias eurytheme* and *Colias philodice* (Lepidoptera, Pieridae): use of olfaction in mate selection.. Ann Entomol Soc Am.

[34] Pliske TE, Eisner T (1969). Sex pheromone of queen butterfly: biology.. Science.

[35] Nishida R, Schulz S, Kim CS, Fukami H, Kuwahara Y (1996). Male sex pheromone of a giant danaine butterfly, *Idea leuconoe*.. J Chem Ecol.

[36] Myers J (1972). Pheromones and courtship behavior in butterflies.. Am Zoologist.

[37] Birch MC, Poppy GM, Baker TC (1990). Scents and eversible scent structures of male moths.. Annu Rev Entom.

[38] VaneWright RI, Boppre M (1993). Visual and chemical signaling in butterflies: functional and phylogenetic perspectives.. Philos Trans R Soc Lond B Biol Sci.

[39] Mustaparta H, Cardé RT, Minks AK (1996). Olfactory coding mechanisms for pheromone and interspecific signal information in related moth species.. Insect pheromone research: new directions.

[40] Costanzo K, Monteiro A (2007). The use of chemical and visual clues in female choice in the butterfly *Bicyclus anynana*.. Proc Biol Sci.

[41] Kemp DJ, Macedonia JM, Ball TS, Rutowski RL (2008). Potential direct fitness consequences of ornament-based mate choice in a butterfly.. Behav Ecol Sociobiol.

[42] Phelan PL, Choe JC, Crespi BJ (1997). Evolution of mate-signaling in moths: phylogenetic considerations and predictions from the asymmetric tracking hypothesis.. The evolution of mating systems in Insects and Arachnids.

[43] Beldade P, McMillan WO, Papanicolaou A (2008). Butterfly genomics eclosing.. Heredity.

[44] Wahlberg N (2006). That awkward age for butterflies: insights from the age of the butterfly subfamily Nymphalinae (Lepidoptera: Nymphalidae).. Syst Biol.

[45] Larsen T (1999). Butterflies of West Africa: origins, natural history, diversity, and conservation..

[46] Condamin M (1973). Monographie du genre *Bicyclus* (Lepidoptera, Satyridae)..

[47] Brakefield PM, Gates J, Keys D, Kesbeke F, Wijngaarden PJ (1996). Development, plasticity and evolution of butterfly eyespot patterns.. Nature.

[48] Brakefield PM (1998). The evolution-development interface and advances with the eyespot patterns of *Bicyclus* butterflies.. Heredity.

[49] Beldade P, Brakefield PM (2002). The genetics and evo-devo of butterfly wing patterns.. Nat Rev Genet.

[50] Brakefield PM, French V, Zwaan BJ (2003). Development and the genetics of evolutionary change within insect species.. Ann Rev Ecol Evol Syst.

[51] Brakefield PM (2006). Evo-devo and constraints on selection.. Trends Ecol Evol.

[52] Schneider MV (1998). Courtship behaviour and description of secondary sexual characters of *Bicyclus anynana*..

[53] Brakefield PM, El Filali E, Van der Laan R, Breuker CJ, Saccheri IJ (2001). Effective population size, reproductive success and sperm precedence in the butterfly, *Bicyclus anynana*, in captivity.. J Evol Biol.

[54] Linn JCE, Roelofs WL (1989). Response specificity of male moths to different blends and dosages of sex pheromone.. Chem Senses.

[55] Leal WS (2005). Pheromone reception.. Topics Current Chem.

[56] Joron M, Brakefield PM (2003). Captivity masks inbreeding effects on male mating success in butterflies.. Nature.

[57] Johansson BG, Jones TM (2007). The role of chemical communication in mate choice.. Biol Rev.

[58] Tillman JA, Seybold SJ, Jurenka RA, Blomquist GJ (1999). Insect pheromones - an overview of biosynthesis and endocrine regulation.. Insect Biochem Mol Biol.

[59] Jurenka RA, Blomquist G, Vogt R (2003). Biochemistry of female moth sex pheromones.. Insect pheromone biochemistry and molecular biology.

[60] Choi MY, Groot A, Jurenka RA (2005). Pheromone biosynthetic pathways in the moths *Heliothis subflexa* and *Heliothis virescens*.. Arch Insect Biochem Physiol.

[61] Beldade P, Rudd S, Gruber JD, Long AD (2006). A wing expressed sequence tag resource for *Bicyclus anynana* butterflies, an evo-devo model.. BMC Genomics.

[62] Shields O (1976). Fossil butterflies and the evolution of Lepidoptera.. J Res Lepid.

[63] Vane-Wright RI (2004). Butterflies at that awkward age.. Nature.

[64] Benton R (2006). On the ORigin of smell: odorant receptors in insects.. Cell Mol Life Sci.

[65] Dess DB, Martin JCJ (1983). Readily accessible 12-I-5 oxidant for the conversion of primary and secondary alcohols to aldehydes and ketones.. Org Chem.

[66] Lundh M, Smitt O, Hedenström E (1996). Sex Pheromone of Pine Sawflies. Enantioselective Lipase Catalysed Transesterification of erythro-3,7-Dimethylpentadecan-2-ol, Diprionol.. Tetrahedron Asymmetry.

[67] Hedenström E, Edlund H, Lund S, Abersten M, Persson D (2002). Synthesis and lipase catalysed stereoselective acylation of some 3-methyl-2-alkanols, identified as sex pheromone precursors in females of pine sawfly species.. J Chem Soc Perkin Trans.

[68] Buser HR, Arn H, Guerin P, Rauscher S (1983). Determination of double bond position in mono-unsaturated acetates by mass spectrometry of dimethyl disulfide adducts.. Anal Chem.

[69] Brooks CJW, Gilbert MT, Gilbert JD (1973). New derivatives for gas-phase analytical resolution of enatiomeric alcohols and amines.. Anal Chem.

[70] Brakefield PM, Reitsma N (1991). Phenotypic plasticity, seasonal climate and the population biology of *Bicyclus* butterflies (Satyridae) in Malawi.. Ecol Entomol.

[71] Svensson GP, Ryne C, Löfstedt C (2002). Heritable variation of sex pheromone composition and the potential for evolution of resistance to pheromone-based control of the Indian meal moth, *Plodia interpunctella*.. J Chem Ecol.

