# Supplementary material for: The Male Sex Pheromone of the Butterfly Bicyclus anynana: Towards an Evolutionary Analysis
Source: PLoS One. 2008 Jul 23;3(7):e2751. doi: 10.1371/journal.pone.0002751 (PMC2447158; doi:10.1371/journal.pone.0002751)
Supplement: Text S1 — Synthesis of 16:Ald and of the stereoisomeric mixtures of 6,10,14-trimethylpentadecane-2-ol. (0.04 MB DOC) [file pone.0002751.s001.doc]

**Supporting information S1:** Synthesis of 16:Ald and of the stereoisomeric mixtures of 6,10,14-trime-15-2-ol.

Commercially available chemicals were used without further purification unless otherwise stated. Chirazyme® L-2, carrier fixed, C2, (Lipase B, from *Candida antarctica*, CAL-B) was obtained from Roche Diagnostics**.** Et_2_O (LiAlH_4_), cyclohexane and EtOAc (CaH_2_) were distilled from the indicated drying agents and stored under argon. The lipase reactions were performed on a shaking board at 25 °C. Samples were taken at intervals and filtered through a pad of MgSO_4_ and rinsed with *n*-heptane before GC-analysis, which was used to assess the conversion of the alcohol in relation to the amount of ester produced. Preparative liquid chromatography (LC) was performed on straight phase silica gel (Merck 60 **,** 230-400 mesh, 0.040-0.063 mm) obtained from Fluka, employing a gradient technique using an increasing concentration (0-100%) of distilled ethyl acetate in distilled cyclohexane as eluent. 6,10,14-trimethylpentadecane-2-ol (1) was transformed to the acetate using a standard procedure (CH_3_COCl, CH_2_Cl_2_). The produced acetate was analyzed by GC (a Varian Star 3400 equipped with a capillary column, β-dex 120, 30 m, 0.25 mm i.d., d_f_=0.25 µm, obtained from Supelco, carrier gas He, 20 psi, split 23/1, temperature program: 80 °C/1 min, then 2 °C/min up to 150 °C). Retention times (min) of the eight stereoisomers: three peaks (1:2:1) 128.6, 129.5 and 130.5 (acetate of (2*S*-1), the acetate of 2*R*-1 elutes at 133.2, 134.2 and 135.2 (1:2:1). Mass spectra were recorded on a Saturn 2000 instrument, (EI-mode) coupled to a Varian 3800 GC. NMR spectra were recorded on a Bruker Avance 500 (500 MHz ^1^H and 125.8 MHz ^13^C) spectrometer using CDCl_3_ as solvent and TMS as internal reference. Optical rotations were determined using a Perkin Elmer 341 polarimeter using a 1 dm cell.

Synthesis of 16:Ald. Hexadecan-1-ol (516 mg, 2.13 mmol) in CH_2_Cl_2_ (15 ml) was added to a solution of Dess-Martin periodinane (1.59 mg, 3.75 mmol) in CH_2_Cl_2_ (15 ml) at room temperature under an argon atmosphere. After 50 minutes, Et_2_0 (50 ml) and aqueous 2M NaOH (30 ml) was added to the reaction mixture and stirred for another 10 minutes. The organic phase was separated, followed by extraction with Et_2_0 (3 x 50 ml). The combined extracts were washed with aqueous 2M NaOH (50 ml), H_2_0 (50 ml) and dried with MgSO_4_. Evaporation of solvent resulted in an orange oil which was purified by LC. The title compound was isolated as a clear faint yellow oil, which crystallised upon standing (464 mg, 1.93 mmol, 91%), with 99.6% purity according to GC. ^1^H NMR (500 MHz; CDCl_3_, Me_4_Si): δ 0.88 (3H, t, *J* = 7.0 Hz), 1.22-1.34 (24H, m), 1.61 (2H, quintet, *J* = 7.3 Hz), 2.42 (2H, dt, *J* = 1.9, 7.4 Hz), 9.77 (1H, t, *J* = 1.9 Hz); ^13^C NMR (125 MHz; CDCl_3_, Me_4_Si): δ 14.14, 22.10, 22.71, 29.18, 29.37, 29.44, 29.60, 29.65, 29.67, 29.69, 29.71, 31.94, 43.95, 203.03; (^1^H and ^13^C NMR were similar to that reported in the literature (Zhao and Zhang, 2007), except for a minor ^1^H resonance displacement and less reported ^13^C resonances); MS (EI) *m/z* (relative intensity): 241 (MH^+^, 4), 240 (M^+^, 2), 222 (6), 194 (7), 180 (3), 166 (8), 152 (7), 138 (17), 124 (25), 110 (27), 96 (69), 81 (87), 67 (100), 57 (48), 41 (50).

Synthesis of 6,10,14-trimethylpentadecane-2-one (**2**). Following the procedure (Sasaerila et al., 2003), phytol (9.2g, 31.1mmol) was oxidized at room temperature in 180ml CH_3_CN and 135ml H_2_O by NaIO_4_ (33g, 0.15mol) and catalytic amount RuCl_3_ (100mg, 0.48mmol). After stirring over night the reaction mixture was extracted with Et_2_0 3x50mL, the combined organic phase was dried with MgSO_4_ and after filtration and evaporation of the solvent an oil was obtained. Purification by LC afforded 8.3g (99% purity) of the title ketone. The ^1^H NMR, ^13^C NMR and mass spectra were identical to those previously reported (Nam et al., 2007; Suga et al., 1989)

Synthesis of 6,10,14-trimethylpentadecane-2-ol (**1**). The reduction of **2** (6.3g, 23.5mmol) by LAH (1.1g, 28.9mmol) in 350ml Et_2_O was quenched after stirring over night by slow addition of 100ml H_2_O. The organic phase was washed with 20mL 2M HCl and 40mL brine, dried with MgSO_4_, filtered and the solvent was evaporated leaving an oil. Purification by LC afforded 4.5g (99% purity) of the title alcohol. The ^1^H NMR, ^13^C NMR and mass spectra were identical to those previously reported (Mori et al., 1991; Nam et al., 2007; Suga et al., 1989)

Synthesis of (2*R*,6*R*/*S*,10*R*/*S*)- 6,10,14-trimethylpentadecane-2-ol (2*R*-1). Following a published procedure for similar alcohols (Hedenström et al., 2002; Lundh et al., 1996). Chirazyme L-2 (CAL-B) (500mg, 65mg/mmol) and molecular sieves (3Å) were added to a solution of the alcohol **1** (2.07g, 7.7mmol) in *n*-heptane (100mL, 13mL/mmol) and stirred for 1h at room temperature, followed by addition of vinyl acetate (13.5mL, 146mmol). After reaching 35% conversion the mixture was filtered and the solid collected was rinsed with *n*-heptane. The solvent was evaporated off and the product acetate of 2*R*-1 was separated from the remaining substrate alcohol by LC, furnishing 0.82g of a colorless oil (99% purity), containing less than 1% of 2*S*-1 (chiral GC).

The isolated acetate 2*R*-1 (0.82g, 2.6mmol) from above was stirred with KOH/MeOH (45mL, 2.4M) at room temperature over night. The reaction was quenched by the addition of H_2_O (30mL) and Et_2_O (30mL). The aqueous phase was extracted with Et_2_O (2x50mL) and the combined organic phases were washed with NaHCO_3_ (2x30mL, aq., sat.), dried (MgSO_4_), filtered and the solvent was evaporated off furnishing the title alcohol 2*R*-1 (0.69g, 98%), >99% pure by GC contaminated only by minor amounts (<1%) of 2*S*-isomers of **1**.

Chirazyme L-2 (CAL-B), 30mg, 66mg/mmol) and molecular sieves (3Å) were added to a solution of the alcohol 2*R*-1 from above (120mg, 0.45mmol) in *n*-heptane (4mL, 8.8mL/mmol) and stirred for 1h, followed by the addition of vinyl acetate (0.7mL, 7.6mmol). After 79% conversion, the mixture was filtered and the solid collected was washed with *n*-heptane. The solvent was evaporated off and the product acetate was separated from the remaining substrate alcohol by LC, furnishing 110mg of a colorless oil (99% purity), with less than 0.01% of 2*S*-1 stereoisomers judged by chiral GC. Reduction of product acetate with LAH in Et_2_O according to previous method furnished after workup the 2*R*-1 alcohol as a colorless oil (87mg, 80%) >99% pure. $\left[\alpha\right]_{D}^{25}$ = −6.7 (c 0.54, *n*-pentane). The ^1^H NMR, ^13^C NMR and mass spectra were identical to those previously reported (Mori et al., 1991; Nam et al., 2007; Suga et al., 1989).

Synthesis of (2*S*,6*R*/*S*,10*R*/*S*)- 6,10,14-trimethylpentadecane-2-ol (2*S*-1). A solution of the recovered alcohol substrate, 2*S*-1, obtained by LC from the first esterification step above was concentrated in vacuo to give 1.37g of a colorless oil (99% purity). This recovered substrate (1.37g, 5.1mmol) in heptane (65mL, 12.7mL/mmol), Chirazyme L-2 (CAL-B, 330mg, 65mg/mmol) and molecular sieves (3Å) were stirred for 1h, followed by the addition of vinyl acetate (8.9mL, 96mmol). After 30% conversion the mixture was filtered and the remaining solid was rinsed with *n*-heptane. The solvent was evaporated off and the remaining alcohol was separated from the ester produced by LC. The fractions containing alcohol were concentrated in vacuo to give 0.97g oil (>99% purity), containing less than 1% of 2*R*-1 isomers (chiral GC of its acetate).

Chirazyme L-2 (CAL-B), 60mg, 132mg/mmol) and molecular sieves (3Å) were added to a solution of the mixture containing mainly alcohol 2*S*-1 (120mg, 0.45mmol) in *n*-heptane (4mL, 8.8mL/mmol) and stirred for 1h, followed by the addition of vinyl acetate (0.7mL, 7.6mmol). After 20% conversion, the mixture was filtered and the solid collected was washed with *n*-heptane. The solvent was evaporated off and the remaining alcohol was separated from the product ester by LC, furnishing 96mg of a colorless oil (>99% purity), containing less than 0.05% of 2*R*-1 isomers (chiral GC of its acetate). $\left[\alpha\right]_{D}^{25}$ = +5.3 (c 3.0, n-pentane). The ^1^H NMR, ^13^C NMR and mass spectra were identical to those previously reported (Mori et al., 1991; Nam et al., 2007; Suga et al., 1989).

References

Hedenström E, Edlund H, Lund S, Abersten M, Persson D (2002) Synthesis and lipase catalysed stereoselective acylation of some 3-methyl-2-alkanols, identified as sex pheromone precursors in females of pine sawfly species. J. Chem. Soc. Perkin Trans. 1: 1810-1817.

Lundh M, Smitt O, Hedenstrom E (1996) Sex pheromone of pine sawflies: enantioselective lipase catalysed transesterification of erythro-3,7-dimethylpentadecan-2-ol, diprionol. Tetrahedron Asymmetry 7: 3277-3284.

Mori K, Harada H, Zagatti P, Cork A, Hall DR (1991) Pheromone synthesis .126. Synthesis and biological activity of 4 stereoisomers of 6,10,14-trimethyl-2-pentadecanol, the female produced sex pheromone of rice moth (*Corcyra cephalonica*). Liebigs Annalen Der Chemie.

Nam T, Rector CL, Kim H, Sonnen AFP, Meyer R, et al. (2007) Tetrahydro-1,8-naphthyridinol analogues of &#x03B1;-tocopherol as antioxidants in lipid membranes and low-density lipoproteins. J. Am. Chem. Soc. 129: 10211-10219.

Sasaerila Y, Gries R, Gries G, Khaskin G, King S, et al. (2003) Sex pheromone components of male *Tirathaba mundella* (Lepidoptera : Pyralidae). Chemoecol.13: 89-93.

Suga T, Ohta S, Nakai A, Munesada K (1989) Glycinoprenols - novel polyprenols possessing a phytyl residue from the leaves of soybean. J.Organic Chem. 54: 3390-3393.

Zhao XF, Zhang C (2007) Iodobenzene dichloride as a stoichiometric oxidant for the conversion of alcohols into carbonyl compounds; two facile methods for its preparation. Synthesis: 551-557.
